# Regulation of phosphate homeostasis by the phosphatonins and other novel mediators

**DOI:** 10.1007/s00467-008-0751-z

**Published:** 2008-08-01

**Authors:** Aisha Shaikh, Theresa Berndt, Rajiv Kumar

**Affiliations:** 1 Division of Nephrology and Hypertension, Department of Internal Medicine, Mayo Clinic Rochester, 200 First St SW, Rochester, MN 55905 USA; 2 Department of Physiology and Biomedical Engineering, Mayo Clinic Rochester, Rochester, MN USA; 3 Department of Biochemistry and Molecular Biology, Mayo Clinic Rochester, Rochester, MN USA

**Keywords:** Phosphate, Vitamin D, Phosphatonins, PTH, Fibroblast growth factors

## Abstract

A variety of factors regulate the efficiency of phosphate absorption in the intestine and phosphate reabsorption in kidney. Apart from the well-known regulators of phosphate homeostasis, namely parathyroid hormone (PTH) and the vitamin D–endocrine system, a number of peptides collectively known as the “phosphatonins” have been recently identified as a result of the study of various diseases associated with hypophosphatemia. These factors, fibroblast growth factor 23 (FGF-23), secreted frizzled-related protein 4 (sFRP-4), fibroblast growth factor 7 (FGF-7) and matrix extracellular phosphoglycoprotein (MEPE), have been shown to play a role in the pathogenesis of various hypophosphatemic and hyperphosphatemic disorders, such as oncogenic osteomalacia, X-linked hypophosphatemic rickets, autosomal dominant hypophosphatemic rickets, autosomal recessive hypophosphatemia and tumoral calcinosis. Whether these factors are true hormones, in the sense that they are regulated by the intake of dietary phosphorus and the needs of the organism for higher or lower amounts of phosphorus, remains to be firmly established in humans. Additionally, new information demonstrates that the intestine “senses” luminal concentrations of phosphate and regulates the excretion of phosphate in the kidney by elaborating novel factors that alter renal phosphate reabsorption.

## Phosphorus homeostasis

*Phosphorus distribution:* Phosphorus plays a critical role in many biological processes, including energy metabolism, cellular signaling through the phosphorylation of proteins and other substances, nucleic acid metabolism, membrane integrity, and bone mineralization. In the male human adult, total body phosphorus is between 15 mol and 20 mol (12.0 g/kg), the majority of which (80–90%) is present in bone in the form of hydroxyapatite [1, 2]. The remainder is present in soft tissues, extracellular fluid and erythrocytes. Soft tissue phosphorus is between 0.1% and 0.3% wet weight tissue (∼59 mmol/kg wet weight muscle). In plasma or serum, phosphorus exists as inorganic phosphate, lipid phosphorus and phosphoric ester phosphorus (concentrations, in millimoles, of each being 0.71–1.36, 2.23–3.13 and 0.86–1.45, respectively). Clearly, the plasma compartment contains only a small fraction of total body phosphorus, and changes in concentrations of inorganic phosphate do not necessarily reflect total body stores of phosphorus.

**Fig. 1:**
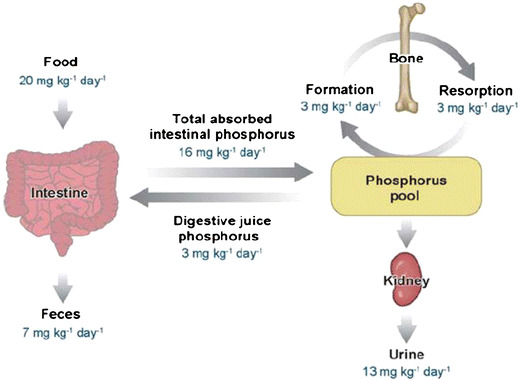
Phosphorus homeostasis in humans. Reprinted with permission [3]

*Phosphorus homeostasis and its regulation:* The amounts of phosphorus moving across various epithelial tissues and organs are depicted in Fig. 1 [3]. Phosphorus is absorbed in the small intestine, predominantly in the jejunum, by both transcellular and paracellular processes (Fig. 1), the former process being mediated by sodium–phosphate type IIb cotransporters [4]. The paracellular pathway for phosphate/phosphorus absorption in the intestine is dependent, in large part, on the concentration of phosphorus present in the intestinal lumen. Increasing amounts of dietary phosphorus are associated with larger amounts of phosphorus absorption, with little evidence of saturation of the process. After entering the extracellular fluid space and circulation, phosphorus enters various tissues, including bone, as a result, at least in part, of the activity of sodium–phosphate type III cotransporters [4, 5]. Plasma inorganic phosphate is filtered at the glomerulus and is reabsorbed in the proximal tubule, largely via the sodium–phosphate cotransporter type IIa [6].

**Fig. 2:**
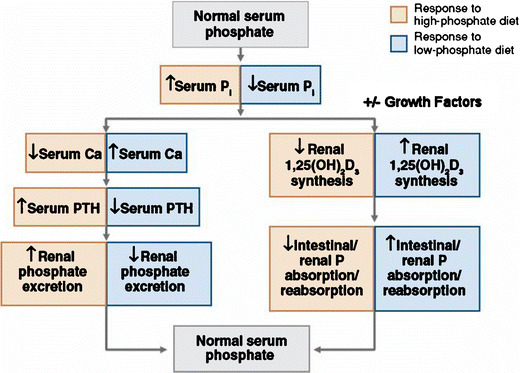
The interaction between parathyroid hormone and vitamin D–endocrine system in the regulation of phosphorus homeostasis

**Fig. 3:**
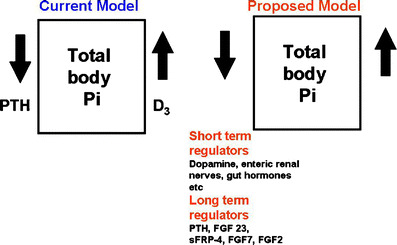
Factors regulating phosphorus homeostasis in humans (*FGF* fibroblast growth factor, *sFRP-4* secreted frizzled-related protein 4)

**Fig. 4:**
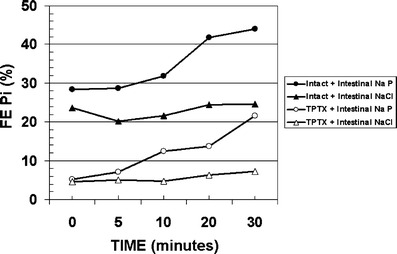
Experimental evidence for the presence of intestinal phosphatonins that mediate changes in renal phosphate excretion following increases in luminal phosphate concentrations in the intestine. Sodium phosphate (*Na P*) or sodium chloride (*NaCl*) was infused into the duodena of rats, and fractional excretion (*FE*) of phosphate was measured at short intervals following the infusion (*TPTX* thyroparathyroidectomized)

**Fig. 5:**
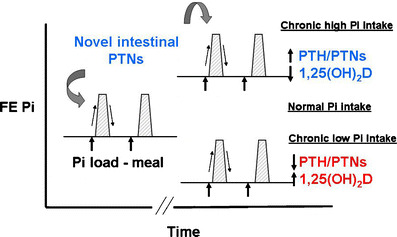
Intestinal phosphatonins mediate changes in the renal fractional excretion (*FE*) of phosphate following the ingestion of meals containing increased amounts of phosphate (*gray hatched areas*). Long-term dietary ingestion of increased amounts of phosphate is associated with increased PTH secretion and reduced 1,25- dihydroxyvitamin D synthesis. The levels of phosphatonins (*PTNs*) may increase following chronic increases in dietary phosphate excretion in some experimental models. Excursions in the fractional excretion of phosphate mediated by the intestinal phosphatonins still occur in the presence of an elevated baseline fractional excretion of phosphate. When phosphorus intake is curtailed, the opposite series of events occurs

The regulation of phosphorus homeostasis is a complex process that involves the interplay between parathyroid hormone and vitamin D endocrine system (Fig. 2) [3, 7]. Phosphorus balance is primarily determined by processes that regulate the efficiency of intestinal phosphorus absorption and renal phosphorus reabsorption. Recent studies have provided evidence that parathyroid hormone (PTH) and vitamin D are not the sole regulators of inorganic phosphate (Pi) homeostasis and have led to the identification of other factors, such as the phosphatonins that contribute to the maintenance of Pi homeostasis. In addition, dietary Pi intake, dopamine, adrenergic activity and blood pH also influence plasma Pi concentrations (Fig. 3) [8]. Recent findings from our laboratory suggest that unique intestinal factors (“intestinal phosphatonins”), released by increases in intestinal luminal phosphate concentrations, alter the renal reabsorption of phosphate (Figs. 4 and 5) [9]. It is likely that these intestinal phosphatonins mediate the short-term changes in the fractional excretion of phosphate observed after ingestion of a meal, and it is likely that they play a role in the short-term adaptation to changes in dietary phosphate. On the other hand, long-term changes in dietary phosphate may be associated with changes in PTH, 1,25-dihydroxyvitamin D and the phosphatonins (see below).

*The phosphatonins and disorders of phosphate homeostasis in humans:* The term “phosphatonin” was coined in 1994 to describe a circulating phosphaturic factor present in the serum of patients with oncogenic or tumor-induced osteomalacia (TIO) [10, 11]. Cai et al. described a patient with TIO in whom the biochemical phenotype of hypophosphatemia, renal phosphate wasting, reduced 1α,25-dihydroxyvitamin D (1α,25(OH)_2_D) concentrations and the osteomalacia resolved after removal of the tumor [10, 11]. X-linked hypophosphatemic rickets (XLH) [12], autosomal dominant hypophosphatemic rickets (ADHR) [13], and autosomal recessive hypophosphatemia (ARHP) [14] are disorders that are phenotypically similar to TIO and which demonstrate the presence of a circulating factor responsible for hypophosphatemia and renal phosphate wasting [14–17]. Conversely, concentrations of one of the phosphatonins, fibroblast growth factor 23, are reduced in patients with tumoral calcinosis (TC), a disorder characterized by hyperphosphatemia, reduced fractional excretion of phosphate and deposits of calcium phosphate in soft tissues [18–24]. Several proteins, such as fibroblast growth factor-23 (FGF-23), secreted frizzled-related protein (sFRP-4), matrix extracellular phosphoglycoprotein (MEPE) and fibroblast growth factor-7 (FGF-7) have been identified as potential phosphatonins and probably play a role in the pathogenesis of some of these disorders [3, 14, 16, 17, 25–28]. Table 1 summarizes the pathophysiology of some of these hypophosphatemic and hyperphosphatemic disorders. A more detailed discussion of each of these peptides and their physiology and pathophysiology follows.

**Table 1:** The pathophysiology of disorders of phosphate homeostasis associated with altered phosphatonin production/circulating concentrations

Clinical disorder	Clinical phenotype	Pathophysiology
Hypophosphatemic disorders		
Tumor-induced osteomalacia (TIO)	Hypophosphatemia, hyperphosphaturia, reduced 1α,25(OH)_2_D concentrations or inappropriately normal 1α,25(OH)_2_D concentrations for the level of serum phosphate, osteomalacia or mineralization defect	Excess of production of phosphatonins—FGF-23, sFRP-4, MEPE, FGF-7 [10, 25, 50, 53, 78]
X-linked hypophosphatemic rickets (XLH)	As in TIO	Mutations in the endopeptidase PHEX that result in increased concentrations of FGF-23, sFRP-4 and MEPE [29, 51, 58, 79]
Autosomal dominant hypophosphatemic rickets (ADHR)	As in TIO	Mutations in the FGF-23 gene that result in the formation of a mutant form of FGF-23 that is resistant to proteolysis [16]
Autosomal recessive hypophosphatemia (ARHP)	As in TIO	Mutations in the gene for DMP-1; associated with elevated concentrations of FGF-23 [14, 17]
Hyperphosphatemic disorders		
Tumoral calcinosis	Hyperphosphatemia, hypophosphaturia, elevated or normal 1α,25(OH)_2_D concentrations, ectopic calcification	Mutations in the genes for Ga1NAc transferase 3 (GALNT3), FGF-23, and Klotho [18–24, 67, 80]. Some patients with GALNT3 and FGF-23 mutations have diminished concentrations of intact FGF-23. The one patient with a Klotho mutation had very high FGF-23 concentrations.
Renal failure	Hyperphosphatemia, hypophosphaturia, reduced 1α,25(OH)_2_D concentrations	Elevated FGF-23 and FGF-7 concentrations

## The biology of phosphatonins

*Fibroblast growth factor-23:* FGF-23 is a secreted, circulating, 32-kDa protein that is predominantly expressed in osteocytes in the bone and in the endothelial cells that line the venous sinusoids of bone marrow and the thymus [29]. FGF-23 null mice have decreased bone mineral density, elevated plasma Pi and 1α,25(OH)_2_D_3_ concentrations and low PTH concentrations [30]. It is difficult to ascertain if the decreased bone mineralization is a direct effect of reduced FGF-23 or a consequence of elevated Pi and 1α,25(OH)_2_D_3_ concentrations. Ectopic calcification in FGF-23 null mice is greatly diminished by either ablation of the vitamin D receptor or by feeding the mice a low phosphate diet, suggesting that elevated calcium and phosphate levels are important in the formation of ectopic mineral deposits [31, 32]. Transgenic mice over-expressing FGF-23 have reduced plasma Pi concentration, phosphaturia and reduced renal sodium phosphate cotransporter [33].

FGF-23 interacts with FGF receptors that belong to type 1 transmembrane phosphotyrosine kinase receptors to elicit a biological response in tissues [34]. Recent studies indicate that FGF-23 also requires Klotho, as a co-factor for receptor activation [34, 35]. In the mouse and human, the klotho/Klotho gene encodes a single-pass membrane protein which has homologies to β-glucosidases [36–39]. Two transcripts formed through alternative RNA splicing are transcribed from the gene and encode a membrane or secreted klotho protein [36]. A circulating and cerebrospinal fluid (CSF) form of klotho is also formed as a result of the cleavage of the membrane-bound form of the protein [36, 40]. Klotho is expressed in several tissues, including the kidney, reproductive tissues and brain [38]. The role of Klotho as a FGF-23 co-receptor is supported by the fact that Klotho-deficient mice have a phenotype similar to that of FGF-23 null mice [37].

**Fig. 6:**
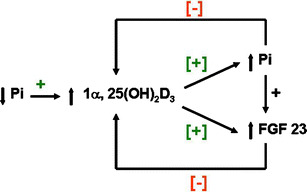
Relationships between changes in Pi, 1α,25(OH)_2_D and FGF-23. Reprinted with permission [3]

The presence of FGF-23 in the circulation of healthy human subjects suggests that it plays a role in the maintenance of Pi homeostasis. In humans, short-term alterations in dietary Pi intake do not alter FGF-23 concentrations [41], and long-term changes in Pi intake have modest or no effect on FGF-23 concentrations [42, 43]. In animals FGF-23 concentrations are suppressed by low Pi diets and are stimulated by high Pi diets [44, 45]. In the short term, however, in rats there are no changes in FGF-23 or sFRP-4 concentrations following increases in intestinal Pi [9]. Serum FGF-23 concentrations increase following the administration of exogenous 1α,25(OH)_2_D_3_ [46], and FGF-23 expression is increased in bone cells following 1α,25(OH)_2_D_3_ treatment [47]. It is possible that the resultant increase in serum Pi concentrations after the administration of 1α,25(OH)_2_D_3_ stimulates the release of FGF-23, which, in turn, reduces serum Pi by promoting phosphaturia (Fig. 6). In hyperphosphatemic states, elevated Pi and FGF-23 concentrations may jointly inhibit formation of 1α,25(OH)_2_D_3_.

*Secreted frizzled-related protein, fibroblast growth factor-7 and matrix extracellular phosphoglycoprotein:* Like FGF-23, sFRP-4 decreases renal Pi reabsorption by reducing sodium phosphate transporters in renal proximal tubules and inhibits formation of 1α,25(OH)_2_D_3_ [25]. FGF-7 inhibits sodium-dependent Pi transport in opossum kidney cells, and anti-FGF-7 antibodies attenuate the phosphate transport inhibition induced by FGF-7 [48]. We have recently shown that FGF-7 is phosphaturic in vivo [49]. MEPE has been shown to increase the fractional excretion of phosphate and to induce hypophosphatemia in vivo [50]. In addition, MEPE inhibits bone mineralization in vitro, and MEPE null mice have increased bone mineralization. Importantly, MEPE does not inhibit 1α,25(OH)_2_D_3_ formation.

We will briefly discuss some clinical disorders in which one or more of the phosphatonins play a key role in the pathogenesis of the disease.

## Role of phosphatonins in clinical disorders

### Tumor-induced osteomalacia

TIO is a syndrome due to the presence of mesenchymal tumors that is associated with hypophosphatemia, hyperphosphaturia, inappropriately low serum 1α,25(OH)_2_D_3_ concentrations and osteomalacia [10]. The resolution of these biochemical and bone abnormalities following tumor removal supports the notion of the presence of a circulating factor (phosphatonin) secreted by the tumor. Numerous reports show elevation of FGF-23 in some, but not all, patients with TIO [33, 51, 52]. Removal of the tumor is associated with reduction in serum FGF-23 concentrations, and there is a temporal association between reduction in FGF-23 concentration and elevation in serum Pi, decrease in renal Pi wasting and increase in 1α,25(OH)_2_D_3_ concentrations [33, 51, 52]. sFRP-4, MEPE and FGF-7 have also been shown to be expressed by tumors associated with TIO [25, 48, 53]. The presence of different phosphatonins in TIO points towards the complex pathogenesis of this clinical condition.

### X-linked hypophosphatemic rickets

Patients with X-linked hypophosphatemic rickets (XLH) manifest phosphaturia, hypophosphatemia and rickets [12, 15, 54]. Parabiosis and kidney cross-transplantation experiments have shown that there is a circulating hypophosphatemic factor present in the serum of Hyp mice (the mouse homolog of human XLH) [55–57]. In XLH, there are mutations of the gene encoding the endopeptidase, PHEX [15]. Patients with XLH have elevated serum concentrations of FGF-23 [51, 58], thereby indicating that PHEX is involved in the processing of FGF-23. Some studies have demonstrated that PHEX is responsible for FGF-23 degradation in vitro [26], whereas others have failed to demonstrate such an effect [59–61].

### Autosomal dominant hypophosphatemic rickets

Autosomal dominant hypophosphatemic rickets (ADHR) is an inherited disorder of Pi homeostasis characterized by phosphaturia, hypophosphatemia, osteomalacia and rickets [13]. The ADHR Consortium identified mutations in the FGF-23 gene that encodes a mutant FGF-23 protein that lacks a normal furin proconvertase site making it resistant to proteolysis [16]. A long-lived stable form of FGF-23 is responsible for the clinical manifestations of this disorder [62].

### Fibrous dysplasia/McCune-Albright syndrome

Fibrous dysplasia is a genetic non-inherited disease caused by somatic activating missense mutations of GNAS 1 that lead to variable clinical features, including polyostotic fibrous dysplasia, with endocrine (precocious puberty, pituitary gigantism, Cushing’s syndrome, thyrotoxicosis) and cutaneous (pigment patches on the skin) abnormalities [63]. Pi wasting is seen in approximately 50% of these patients and is associated with defective bone mineralization. One study demonstrated that FGF-23 concentration was elevated in patients with hypophosphatemia but was not increased in patients with normal Pi concentrations [64]. It is possible that the fibrous dysplastic tissue secretes FGF-23, and that serum FGF-23 concentrations are reflective of the disease burden in these patients.

### Tumor calcinosis

Patients with tumor calcinosis (TC) manifest hyperphosphatemia, reduced renal Pi excretion and elevated 1α,25(OH)_2_D_3_ concentrations [65]. Three different types of mutations account for this syndrome. The first type occurs in the gene Ga1NAc transferase 3 (GALNT3), which encodes a glycosyltransferase responsible for initiating mucin-type O-glycosylation [18]. Some patients with this syndrome have low concentrations of intact FGF-23 but high concentrations of FGF-23 fragments. It has been hypothesized that these FGF-23 fragments lack biological activity, and, therefore, the clinical picture is consistent with what would be seen with low intact FGF-23 concentrations. In vivo infusion studies with FGF-23 fragments, however, have shown that carboxyl-terminal fragments are biologically active [66]. At present, there is uncertainty as to the precise mechanism by which GALNT3 mutations cause the syndrome. The second class of mutations responsible for TC occur in the gene encoding FGF-23 [22, 24]. This mutation results in defective processing of FGF-23 and its retention in the Golgi apparatus. Failure to secrete FGF-23 results in low serum concentrations of FGF-23, which, in turn, results in hyperphosphatemia due reduced renal Pi excretion. A third class of mutations responsible for TC occurs in the gene for Klotho [67], which encodes the co-receptor for FGF-23.

### Renal failure

Serum FGF-23 concentrations are elevated in patients with chronic renal failure (CRF), and the increase in FGF-23 correlates with the decline in glomerular filtration rate [68–70]. Elevated plasma Pi seen in renal failure could increase FGF-23 production, although it is possible that reduced clearance of the peptide might also be responsible. Whether or not the elevated serum FGF-23 concentrations found in chronic renal insufficiency are sufficient to correct the hyperphosphatemia of early and advanced CRF is not completely clear. Elevated FGF-23 could play a role in the suppression of 1,25(OH)_2_D production and the development of secondary hyperparathyroidism. The role of FGF-23 in renal osteodystrophy has not been established. Indeed, a recent study shows no effect of FGF-23 on bone histology in end-stage renal disease [71]. Recently, Fliser et al. [72] showed a correlation between increased FGF-23 concentrations and the progression of chronic renal failure in subjects with mild-to-moderate chronic renal disease, suggesting that FGF-23 may play a role in the progression of renal failure. It should be noted, however, that the number of other variables such as the calcium X phosphate product, parathyroid hormone, and vitamin D usage also correlated with progression in the subjects. Finally, there are no data available at present suggesting a direct role of FGF-23 on renal fibrogenesis.

### Post-transplant hypophosphatemia

In some patients following transplantation, persistent hypophosphatemia is noted, despite relatively modest increases in concentrations of circulating parathyroid hormone [68, 70, 73, 74].

In such subjects, FGF-23 concentrations have been noted to be elevated, and it is possible that elevations in the concentrations of this growth factor are responsible for the hypophosphatemia seen in this situation.

### Elevations in FGF-23 in patients with tumors

In patients with humoral hypercalcemia of malignancy, and with metastatic ovarian cancer, FGF-23 concentrations are elevated without significant hypophosphatemia [75–77]. This would suggest that tumors produce FGF-23, and that FGF-23 concentrations must reach a certain significant threshold in order to increase phosphate excretion in the kidney.

In conclusion, phosphatonins play a vital role in the pathogenesis of a wide array of disorders. The presence of several phosphatonins and their differential effects affirm the complexity of Pi regulation in both normal and disease states. Future studies are needed to better understand the role of these proteins.
